# A Mysterious DRESS Case: Autoimmune Enteropathy Associated with DRESS Syndrome

**DOI:** 10.1155/2017/7861857

**Published:** 2017-11-26

**Authors:** Abimbola Adike, Vaishnavi Boppana, Dora Lam-Himlin, Melissa Stanton, Steven Nelson, Kevin C. Ruff

**Affiliations:** ^1^Division of Gastroenterology and Hepatology, Mayo Clinic, Scottsdale, AZ, USA; ^2^Department of Laboratory Medicine and Pathology, Mayo Clinic, Scottsdale, AZ, USA

## Abstract

Drug Reaction with Eosinophilia and Systemic Symptoms (DRESS) is a rare but potentially life-threatening cutaneous hypersensitivity reaction characterized by extensive mucocutaneous eruption, fever, hematologic abnormalities, and extensive organ involvement. Here, we present a case of a young woman with DRESS syndrome following exposure to vancomycin with renal, cutaneous, and gastrointestinal involvement. To the best of our knowledge, this is the first case description in the literature of DRESS of the gastrointestinal tract with autoimmune enteropathy.

## 1. Introduction

Drug Reaction with Eosinophilia and Systemic Symptoms (DRESS) is an uncommon, potentially life-threatening adverse drug reaction. It is also known as drug-induced hypersensitivity reaction, drug-induced delayed multiorgan hypersensitivity syndrome, or drug rash with eosinophilia and systemic symptoms and was previously described in earlier literature as drug-induced pseudolymphoma [1] or anticonvulsant hypersensitivity syndrome as anticonvulsants are the most frequently implicated drugs. Many other drugs including allopurinol, sulfonamides, minocycline, olanzapine, vancomycin, and dapsone have been reported as causative drugs in DRESS [2, 3].

Cases of DRESS can be described as definite, probable, or possible, using the European Registry of Severe Cutaneous Adverse Reaction (RegiSCAR) scoring system which includes clinical features, organ involvement, and disease duration [4]. The most common organ involvement in DRESS is the liver and kidneys, but many other organs can also be involved including the gastrointestinal tract [4].

Case reports represent a major source of information on DRESS in the literature [4]. However, there is a relative paucity of reports on the gastrointestinal manifestations of DRESS. Our case report is the first to describe an evolvement of DRESS to autoimmune enterocolopathy.

## 2. Case Report

A 29-year-old woman with a history severe symptomatic pectus excavatum underwent a repair. She otherwise had no significant past medical history. The surgery was complicated by a* Staphylococcus caprae *right chest pocket infection which was treated with vancomycin. Three weeks following initiation of vancomycin, the patient presented with a diffuse, eruptive, exfoliative, and nonpruritic whole body rash with nonerosive mucositis. She also reported large volume, nonbloody, postprandial diarrhea with up to 4 to 5 loose stools a day. On presentation, the patient was found to have a temperature of 102 F and was hemodynamically stable.

**Figure 1:**
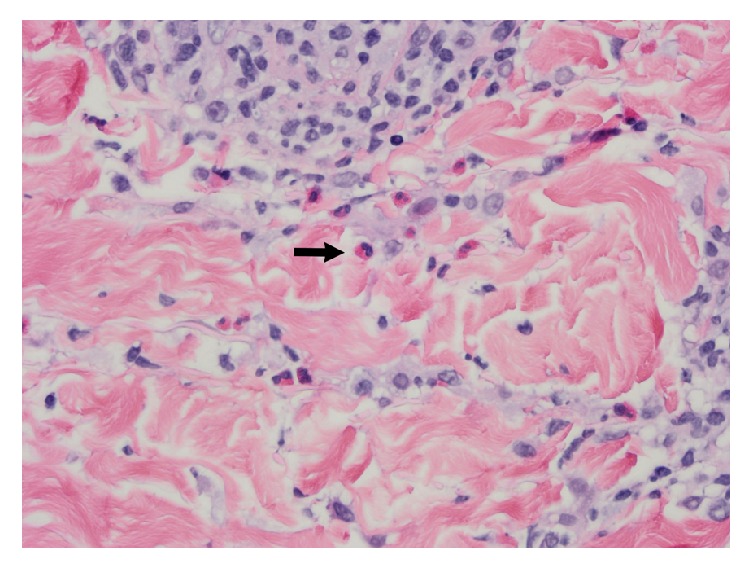
Skin biopsy demonstrating a perivascular and interstitial lymphohistiocytic infiltrate with numerous eosinophils (arrow). Hematoxylin-eosin stain, 400x.

Laboratory values showed white blood cell count 34.5 × 10^9^ with 22% eosinophilia, hemoglobin 8.1 g/dl, creatinine of 2 mg/dl from a prior baseline of 0.7 mg/dl, and bicarbonate 16 mmol/L with a normal anion gap. Blood cultures, acute hepatitis panel, and a stool lactoferrin were negative, as was a complete panel of enteric infectious stool studies including bacterial, viral, and parasitic pathogens. Cytomegalovirus (CMV) DNA checked at weeks 1 and 3 following onset of diarrhea was undetectable and at week 4, CMV DNA was 13, 300 IU/mL. At week one following onset of diarrhea, the following viruses were also tested: human herpes virus 6 (HHV) DNA was positive, and Epstein Barr virus (EBV) DNA was <2000 IU/mL. Gastrin was 100 pg/ml. 24-hour urine 5-hydroxyindoleacetic acid and vasointestinal peptide were normal. Stool alpha-1-antitrypsin was normal. Celiac gene testing was positive for DQ8. Tissue transglutaminase A IgA level was <1.2 U/ml with an IgA of 292 mg/dl. A skin biopsy of the right upper extremity (Figure 1) showed prominent papillary dermal edema with spongiosis and a dense lymphohistiocytic infiltrate with eosinophils which were felt to be consistent with a drug reaction. Computed tomography of the abdomen with oral contrast showed diffuse mesenteric edema without obstruction.

**Figure 2:**
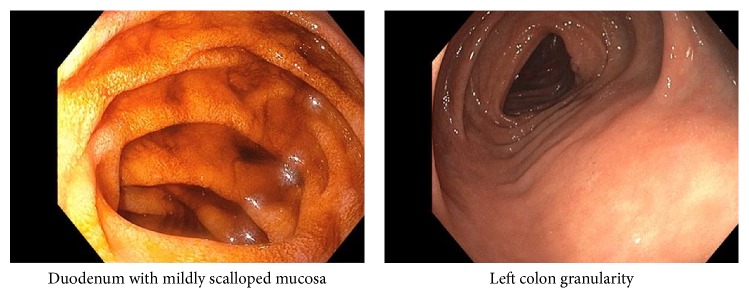
Localized mildly scalloped mucosa in the 2nd portion of the duodenum and granularity in the left colon.

**Figure 3:**
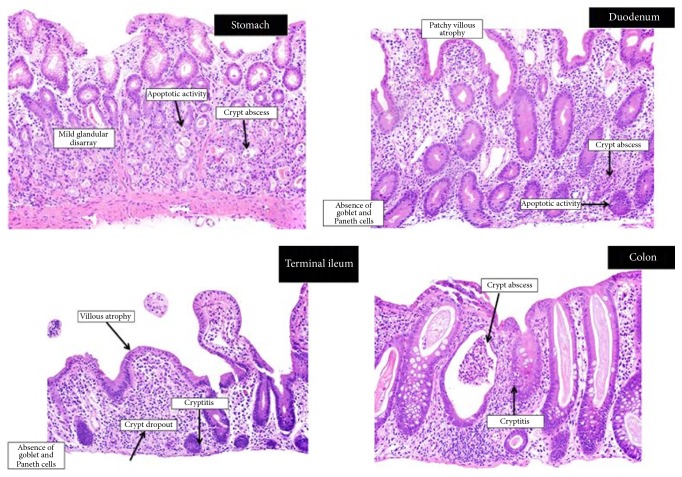
Biopsies of the stomach show loss of parietal cells, mild glandular disarray, acute inflammation, and apoptotic activity (hematoxylin and eosin, original magnification 200x); biopsies of the duodenum show absence of goblet cells and Paneth cells (hematoxylin and eosin, original magnification 200x); biopsies of the terminal ileum are similar to the duodenum. There is an absence of goblet cells and Paneth cells. This field shows crypt dropout as well (hematoxylin and eosin, original magnification 200x); biopsies of the colon show cryptitis and crypt abscess with minimal crypt distortion.

Upper endoscopy showed a normal appearing esophagus and stomach with localized mildly scalloped mucosa in the 2nd portion of the duodenum (Figure 2). Colonoscopy showed granularity in the left colon and a white thin exudate in the entire examined colon (Figure 2). The terminal ileum was normal. Histopathological evaluation of the gastrointestinal tract revealed loss of parietal cells, mild glandular disarray, acute inflammation, and apoptotic activity in the stomach (Figure 3, hematoxylin and eosin, original magnification 200x) and patchy villous atrophy and absence of goblet cells and Paneth cells in the duodenum with crypt abscesses and apoptosis (Figure 3, hematoxylin and eosin, original magnification 200x); and in the terminal ileum, as in the duodenum, there was also an absence of goblet and Paneth cells, with crypt dropout (Figure 3, hematoxylin and eosin, original magnification 200x). Colon biopsies showed cryptitis and crypt abscess with minimal crypt distortion. Although the surface epithelium appeared attenuated, the deeper crypts had goblet cells present (Figure 3, hematoxylin and eosin, original magnification 200x). CMV immunohistochemical stains performed on upper and lower GI biopsies were negative.

The patient subsequently developed severe protein-calorie malnutrition requiring total parenteral nutrition. Vancomycin was felt to be the etiology of the drug reaction with peripheral eosinophilia and systemic symptoms (DRESS) with gastrointestinal, cutaneous, and renal involvement. Vancomycin was discontinued immediately on suspicion that this was a case of DRESS syndrome. The diarrhea responded to high dose intravenous steroids at 2 mg/kg. She was also treated with ganciclovir for CMV viremia.

**Figure 4:**
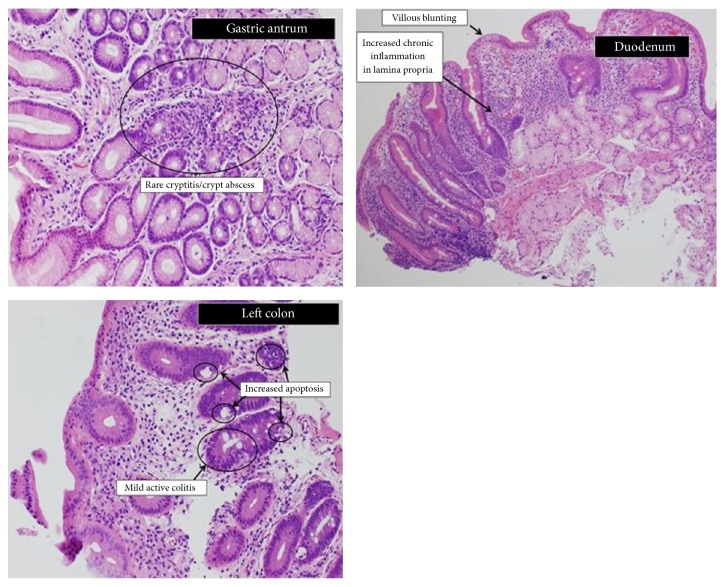
Overall acute inflammation and fewer architectural changes than that reported in this patient's prior biopsy. The gastric biopsies show increased lamina propria chronic inflammation with only rare crypts with cryptitis and crypt abscesses. Oxyntic glands are in abundance in the gastric body biopsies; The duodenum shows both goblet cells and Paneth cells and mild villous blunting, in contrast to the marked changes seen previously; The colon shows patchy active colitis, increased apoptosis, and marked loss of goblet cells.

At followup two months later, the patient was weaned off parenteral nutrition and intravenous corticosteroids, now at an oral dose of 1 mg/kg, and tolerating a regular diet with complete resolution of diarrhea. Repeat upper and lower endoscopy revealed duodenal mucosa was endoscopically normal. Histopathological evaluation overall (Figure 4) revealed less acute inflammation and fewer architectural changes when compared to her prior biopsies but was suggestive of an evolving autoimmune enterocolopathy with villous blunting of the duodenum and absence of goblet and Paneth cells in the colon. Anti-enterocyte IgA and IgG showed linear periapical staining of enterocytes consistent with the presence of anti-enterocyte antibodies.

## 3. Discussion

First coined in 1996, DRESS is a life-threatening adverse drug reaction [1, 2]. DRESS typically presents with fever, rash, lymphadenopathy, eosinophilia, and multiorgan involvement. It has a period of latency of about two to eight weeks from drug exposure [3], with one prospective series reporting a median time interval after drug exposure of 22 days [4]. DRESS typically has a relatively later onset of presentation than other drug eruptions [4]. Our patient presented three weeks after drug exposure to vancomycin with fever, a morbilliform exfoliative rash involving more than 50% of her total body surface area, and peripheral eosinophilia, with acute kidney injury and large volume nonbloody diarrhea. After exclusion of other potential causes including hepatitis, it was felt that this was a definite case of DRESS syndrome from vancomycin exposure, with a RegiSCAR score of 6 (fever, peripheral eosinophilia, skin rash involving more than 50% of the total body surface area, multiorgan involvement, and disease duration of more than 15 days) [4]. In recent years, there has been an increase in the number of DRESS attributed to vancomycin [5]. Despite prompt withdrawal of the culprit drug, DRESS can have a relapsing-remitting course [6]. Retrospective studies have reported a mortality rate of 5–10% [7].

The pathogenesis of DRESS is not well understood. Drug specific activation of T-cells and herpesvirus reactivation has been suggested to be the pathogenesis of DRESS with expansion of regulatory T-cells leading to virus reactivation [3]. It is also possible that antiviral T-cells cross react with the culprit drug [3]. HHV 6, CMV, EBV, and other herpesviruses have been implicated in the pathogenesis of DRESS. The presence of HHV 6 has been said to predict a relapsing course of DRESS with worse prognosis [8, 9].

In addition to a positive HHV 6, our patient had florid CMV viremia which was treated with antiviral therapy. CMV viremia was the unlikely culprit of our patient's symptoms for the following reasons: (1) initial CMV DNA was tested twice and were undetectable at the onset of symptoms. (2) CMV immunohistochemical stains on all upper/lower GI biopsies were negative prior to treatment of CMV viremia. (3) The histopathological features of her upper/lower GI biopsies were not felt to be consistent with CMV enteritis or colitis. (4) The patient's skin biopsies were consistent with a lymphohistiocytic infiltrate with eosinophils which were felt to be consistent with a drug reaction, and not related to a viral infection. For these reasons, we believe that viral reactivation eventually occurred during the course of illness in this patient and was not solely responsible for her constellation of symptoms. Antiviral agents are not routinely used to treat DRESS in the absence of evidence for viral reactivation [8].

Clinical manifestations of DRESS involving the GI tract can include nausea, vomiting, bloody, and nonbloody diarrhea [2]. Histopathological gastrointestinal manifestations of DRESS can range from active colitis with cryptitis, erosion, and ulceration, to eosinophilic infiltrate in the lamina propria, to chronic inflammation with architectural distortion [2]. Prompt withdrawal of the offending drug is the most critical part of management of DRESS [10]. Systemic steroids (0.5 mg/kg to 2 mg/kg) with supportive management are the mainstay of therapy in these patients with a gradual slow taper over a period of 3–6 months while monitoring clinical and laboratory data [10].

While most patients will have complete recovery from DRESS after withdrawal of the offending agent [10], long-term sequelae of DRESS have been reported [11]. In particular, autoimmune diseases have been reported in retrospective cohort studies, including Grave's disease, autoimmune hemolytic anemia, lupus, alopecia areata, and type one diabetes [11–13]. DRESS has also been associated with hemophagocytic lymphohistiocytosis (HLH) [2, 14]. Previously, Swanson et al. reported a fatal case report of a young woman who developed DRESS from antiepileptic drugs with subsequent HLH diagnosed about 5 months after her initial presentation; with endoscopy biopsies that revealed marked villous atrophy in the duodenum with absence of goblet and Paneth cells in the duodenum and colon [2]. Like this patient, our patient also had no eosinophils on initial or subsequent gastrointestinal biopsies. However, her histopathological evaluation of the small bowel showed villous atrophy, apoptotic bodies, and complete absence of goblet and Paneth cells; biopsies of the stomach showed an unusual pattern of injury with chronic inflammation, glandular abscesses, and parietal cell atrophy while in the colon, there was increased apoptosis and marked loss of goblet cells and Paneth cells. Taken together, these histopathological features are reminiscent of autoimmune enterocolopathy [15]. Autoimmune enteropathy is characterized by refractory diarrhea with malabsorption, histologic changes on small intestinal biopsy, and the need for immunosuppressive treatment [15]. The diagnostic criteria for autoimmune enteropathy include chronic diarrhea, malabsorption, small bowel villi blunting, deep crypt lymphocytosis, and increased apoptotic bodies with minimal intraepithelial lymphocytosis and exclusion of other causes of villous atrophy. Our patient met all of these criteria. Although our patient also had a positive anti-enterocyte antibody, anti-enterocyte or anti-goblet cell antibodies are supportive but are not necessarily diagnostic of autoimmune enteropathy [16]. Treatment includes budesonide, prednisone, and other immunosuppressants such as 6-mercaptopurine, azathioprine, mycophenolate mofetil, or cyclosporine [15]. There is very limited data on prognosis of autoimmune enteropathy [15]. To the best of our knowledge, this is the first case report in the literature that describes an evolving autoimmune enterocolopathy from DRESS syndrome.

In conclusion, DRESS is a poorly understood systemic drug eruption which can have significant mortality. While most patients completely recover from DRESS syndrome, a few patients can develop autoimmune diseases. This is the first case report described in the literature of autoimmune enterocolopathy associated with DRESS syndrome.
